# Optimizing Monitoring Frequency During Blood Transfusions: A Review of Guidelines and a Retrospective Cohort to Define a 7-Point Schedule

**DOI:** 10.3390/nursrep15120421

**Published:** 2025-11-28

**Authors:** Siti Zubaidah Mordiffi, Su Wei Wan, Shir Ying Lee, Karen Lim, Poh Chi Tho, Siew Ping Lang, Seri Sastika Ramli, Jerrald Lau, Ker Kan Tan, Karen Wei Ling Koh

**Affiliations:** 1 Nursing Department, National University Hospital, Singapore 119228, Singapore; poh_chi_tho@nuhs.edu.sg (P.C.T.); karen_wl_koh@nuhs.edu.sg (K.W.L.K.); 2 JBI Singapore National University Hospital Nursing Centre, Singapore 119074, Singapore; siew_ping_lang@nuhs.edu.sg; 3 Department of Surgery, Yong Loo Lin School of Medicine, National University of Singapore, Singapore 119228, Singapore; suwei.w@nus.edu.sg (S.W.W.); jerrald.lau@nus.edu.sg (J.L.); ker_kan_tan@nuhs.edu.sg (K.K.T.); 4 Department of Haematology-Oncology, National University Cancer Institute, Singapore 119074, Singapore; shir_ying_lee@nuhs.edu.sg; 5 Blood Transfusion Services, Department of Laboratory Medicine, National University Hospital, Singapore 119074, Singapore; karen_pl_lim@nuhs.edu.sg; 6 Nursing Division, National University Cancer Institute, Singapore 119074, Singapore; seri_sastika_ramli@nuhs.edu.sg; 7 Saw Swee Hock School of Public Health, National University of Singapore, Singapore 117600, Singapore; 8 Division of Surgical Oncology, National University Cancer Institute, Singapore 119228, Singapore

**Keywords:** blood transfusion, transfusion reactions, vital signs monitoring, transfusion practice optimization, evidence-based nursing, clinical guidelines, patient safety, healthcare efficiency, nursing

## Abstract

**Background/Objectives:** Vital signs monitoring during blood transfusion is important but inconsistently practiced across contexts. This paper aimed to consolidate the available evidence to determine the optimal monitoring frequency that balances efficiency and safety in clinical practice. **Methods:** Evidence was gathered through a literature review, review of international guidelines, investigation of local practices and analysis of study institution’s retrospective data on transfusion reaction patterns. Expert opinions were consulted on the proposed changes, prior to the pilot feasibility study. **Results:** The majority of the reviewed guidelines and practices monitored vital signs at three time-points: before transfusion, 15 min after initiation and upon completion. However, study hospital data revealed that transfusion reactions predominantly occurred within the first two hours, particularly among males aged 50–70 who received red packed cells in the oncology wards and had abnormal pre-transfusion vital signs. Thus, the original 10-point frequency practiced by the study hospital was modified to seven time-points instead of the widely adopted three time-points: prior to blood transfusion; 15 min after commencement; 30 min at the forty-fifth minute; hourly thereafter until completion; and within 1 h post-transfusion. **Conclusions:** Despite existing guidelines recommending only three vital signs monitoring time-points, institutional data suggests that using seven time-points is optimal to minimize missed transfusion reactions while preventing an unnecessary workload, balancing patient safety and operational efficiency. These proposed revisions will be evaluated through an upcoming pilot trial to assess their feasibility and the impact on patient outcomes.

## 1. Introduction

Transfusion of blood and blood components such as whole blood, packed red blood cells, plasma, platelets and cryoprecipitate are typically administered to treat hemorrhage and blood component deficiencies arising from various diseases [1]. In large countries like the United States [2], these procedures are vastly prevalent, easily reaching 10.764 million encounters in a single year. Even in small countries like Singapore, up to 119,720 units of blood are administered yearly [3]. These occurrences are likely to become more common, with many populations aging worldwide and treatment complexity increasing, especially for cancer [4].

Vital signs monitoring throughout transfusion is critical for detecting early signs of transfusion-induced reactions that range from mild allergic reactions or febrile symptoms to severe and life-threatening acute hemolysis, anaphylaxis, circulatory overload, transfusion-transmitted sepsis and acute lung injury [5]. Although seemingly basic and routine, these parameters alert the clinical team to possible impending complications that can be acted upon and prevented from worsening [6]. According to Crookston and team [7], hemodynamic deviations within the acceptable margins of 0.5 degrees Celsius body temperature, five respiratory revolutions per minute, 10 beats per minute for heart rate and 20 mmHg for blood pressure are deemed normal. Readings are thus considered abnormal if they fall outside of these stipulated ranges or manifest clinically as hives, itching, fever, chills, hypotension or dyspnea [8].

Hospitals worldwide have practiced frequent vital signs monitoring for many years without much empirical basis. While such routines ensure patient safety, excessive checks can be time-consuming and resource-intensive to perform punctually in surgical, cardiac and oncology units, where transfusions are numerous and frequent and patient acuity is high [9]. In addition, poor staffing is a longstanding issue that has been shown to lead to missed observations, increased adverse events and a prolonged length of stay in patients [10]. Given the demands for safe practice amidst scarce resources, it was necessary to review and re-evaluate the existing literature, international guidelines, local practices and institutional data to update the current blood transfusion vital signs monitoring protocol to keep abreast with evidence-based practice. Hence, this paper aimed to determine the monitoring frequency that was appropriate for practice, based on the collated evidence and institutional findings.

## 2. Methods

This study utilized a multi-phased approach to examine blood transfusion monitoring practices. A review of the literature was conducted using the search terms “blood transfusion”, “vital signs monitoring” and “frequency”. Thereafter, the data obtained were distilled to decipher the onset of side effects and the period of vigilance required to detect these adverse events. International guidelines were referenced, and local practices were examined.

A retrospective analysis was conducted on all transfusion encounters and reaction episodes recorded at our 1200-bed academic tertiary hospital. Data for this study was extracted from our Blood Bank’s data management system from January 2021 to December 2022. This system compiles information on all transfusion encounters and reaction episodes through established protocols within our institution. When healthcare providers suspect a transfusion reaction, they initiate a process through the EPIC electronic health record system, which prompts the completion of a transfusion reaction form and/or sending a urine (urinalysis dipstick (Beckman Coulter, iChem Velocity Urinalysis dipstick, Brea, CA, USA) for presence of blood) and blood sample (Blood group and Coombs test (QuidelOrtho, San Diego, CA, USA) for ABO incompatibility) for laboratory analysis. The time of commencement for the blood transfusion, time and characteristics of the transfusion reaction and time the transfusion was stopped or completed are documented in the transfusion reaction form. Blood bank staff then access these forms and conduct a thorough review of the patient’s electronic medical records, including clinical notes, to gather detailed information about the suspected reaction. Time-to-reaction was derived from the time of commencement of blood transfusion to time of transfusion reaction, recorded in the transfusion reaction form. To ensure inclusivity, all recorded transfusion encounters and reaction episodes that occurred in all areas of the hospital within the specified time frame (2021 to 2022) were included in the analysis, regardless of the severity or type of reaction. Descriptive statistics were used to summarize and present the data to provide an overview of reaction patterns within our institution during the two-year study period. Institutional approval was obtained and approved as ‘Review Not Required’ by the study institution’s research office for the analysis, access and use of anonymized blood transfusion data—NUH-RNR-2025-0025. An exemption was granted, on the basis that this is a retrospective review of the data. Thus, no interventions were initiated, and only de-identified data were used during the analysis.

## 3. Results

### 3.1. Literature Critique

The search did not find any official international guidelines on blood transfusion monitoring, and scientific research focusing on vital signs and their specifics was limited and vague. In a scoping review that examined nursing care for adults receiving blood transfusions, only 10 out of 19 studies (52.6%) mentioned vital signs as a key component [6]. Additionally, there was a lack of high-level interventional and comparative evidence justifying the adoption of one monitoring frequency over another. Practices varied across studies without clear reasons being given for the decisions. For example, one Brazilian study recommended monitoring the patient for the first 10 min, with vital signs being checked at 30 min, 1 h, 2 h, 3 h and 4 h [11], while others from the same country suggested continuous monitoring for adverse reactions during the entire transfusion [12,13]. Some provided brief, generic instructions to check vital signs at the beginning and end of the transfusion, or before, during and after [14,15,16,17]. A 2021 Ghanaian study advised baseline checks 30 min before transfusion [18], while an Egyptian study recommended hourly vital signs throughout the procedure [19]. Additionally, little information was available about the onset of common transfusion reactions, aside from them being classified as either acute (during or within 24 h of transfusion) or delayed (days to weeks after) [20].

### 3.2. Country-Specific Guidelines

Ten online resources, including a review, retrospective study and country-specific protocols that detail the intervals for documenting vital signs, were identified. A general consensus was established on obtaining baseline parameters: rechecking 15 min after initiation and at the end of the transfusion. However, there was no clear guidance regarding surveillance between the first 15 min and the end of the transfusion, which typically lasts around 4 h. Based on the findings, it seems that only guidelines from New Zealand stipulate subsequent 30 min to hourly assessments after the first check from the start of the transfusion [21,22]. Those from the United Kingdom [23,24,25] and the United States [26,27,28] did not mandate intermediary time-points, while Health District Australia [29] advised practitioners to adhere to organizational policies. Table 1 summarizes these findings.

### 3.3. Local Guidelines

Practices across local healthcare institutions in Singapore were studied and the varying intervals adopted by the respective sites are summarized in Table 2. All acute hospitals in Singapore monitor vital signs pre-transfusion, 15 min after commencement and post-transfusion. However, monitoring frequencies in the first and second hour diverged. Three hospitals (Study Hospital, Hospitals E and F) were monitored four times in the first hour and twice in the second hour, followed by hourly until the end of transfusion. Conversely, some hospitals have cut back on first-hour checks; three hospitals (B, C and G) only monitored 15 min after starting, then hourly until the transfusion ended. Hospital A, following an initial 15 min check, conducted regular visual observations of patients without performing routine vital sign measurements after the first hour.

### 3.4. Insights from Retrospective Institution Database Assessment

Retrospective data extracted from the 1200-bed study hospital were analyzed. This was undertaken to obtain a comprehensive understanding of transfusion reaction incidence rates and the time of occurrence during the transfusion to inform the appropriate vital signs monitoring interval to be adopted.

Between 2021 and 2022, a total of 48,362 units of blood products were transfused, comprising red blood cells (32,550 units), platelets (9424 units), fresh frozen plasma (2777 units) and cryoprecipitate (1013 units). There were 72 (0.15%) episodes of transfusion-related reactions (Table 3). The majority of those with such reactions were in oncology wards and received red blood cells (n = 40, 55.6%).

Transfusion reactions were formally reviewed by a pathologist who classified the type of reaction according to ISBT [32] standard definitions, using information about clinical signs and symptoms and laboratory investigations, for the purpose of documentation to patients’ charts and for national hemovigilance reporting. Our study’s definitions of non-serious include those reactions that typically only require symptomatic treatment and would only have been classified as this type of reaction if clinically mild. They would correspond to the ISBT Grade 1 (non-severe) definition, whereas serious reactions are clinically more severe, require in-patient hospitalization or prolongation of hospitalization, or necessitate medical intervention or intensive care to prevent serious threats to health. These correspond to ISBT Grade 2 (severe) and Grade 3 (life-threatening) reactions.

Patient characteristics associated with blood transfusion reactions were similar among the non-serious (n = 57) and serious (n = 15) cases (Table 4). Males predominated in both groups (60%). The most affected age groups were 61–70 years and 51–60 years, for non-serious and serious reactions, respectively. Oncology departments accounted for 29.8% of non-serious and 46.7% of serious reactions. Red blood cells were the most commonly transfused product in both groups (56.1% non-serious, 53.3% serious). Non-serious reactions typically occurred in the second hour (33.3%), while serious reactions were more common within the first hour (26.7%). Pre-transfusion fever was more prevalent in non-serious reactions, while pre-transfusion hypotension was only observed in serious reactions.

**Figure 1:**
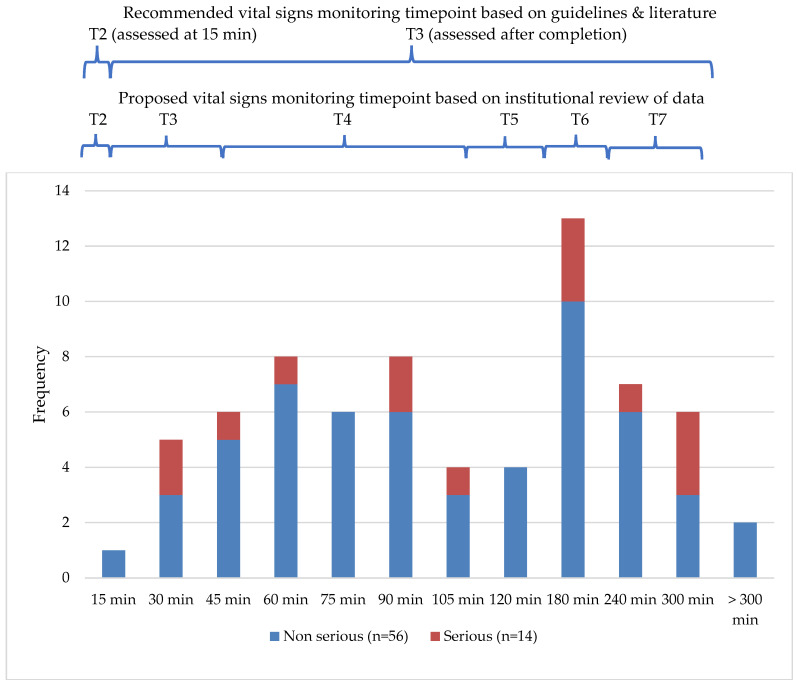
Time to blood transfusion reaction (2021–2022). Note: T1 is not reflected in the graph, as it is prior to transfusion.

Reaction symptoms predominantly manifested within the first two hours of transfusion (n = 42, 60.0%), while the more severe reactions typically occurred at the third hour of transfusion or in the immediate hour post-transfusion (Figure 1). This means that applying the universal minimum vital signs measurement only once, 15 min after the initiation of blood transfusion and at its completion (i.e., 4th hour), could possibly result in at least 67 reaction events being missed or detected late (95.7.1%). It is also important to consider that minimally communicative, unconscious or pediatric patients may not be able to verbalize discomfort or symptomatic reactions, and hence, deviations in vital signs may be the clue to untoward effects.

## 4. Discussion

### 4.1. Proposed Changes and Justifications

Although most international guidelines and local practices recommend at least three vital signs monitoring time-points (before, at 15 min and at completion of transfusion), the data used to gauge the appropriate interval length supports the accommodation of at least seven time-points: (i) pre-transfusion; (ii) at 15 min after initiation; (iii) at 45 min (30 min interval) (iv–vi) at 105, 165 and 225 min (hourly intervals) (based on 4 h transfusion); and (vii) 1 h post-transfusion. This proposed seven time-point monitoring schedule for blood transfusions aligns with Cortez-Gann et al.’s [28] recommendation of “a regular pattern of vital sign monitoring spanning the entire transfusion”, based on a mean time-to-reaction of 1.5 h and a mean time of severe reactions of 2 h or within 8 h of transfusion. Moreover, their study also saw only a small percentage of reactions (15%) occurring in the first 15 min—this finding corroborates with ours in justifying the need for hourly checks up to the 4 h mark to avoid missed identification of a substantial number of transfusion reactions. That said, the decision to conduct a check at 45 min post-initiation instead of 30 or 60 min was made primarily based on two reasons: firstly, this time-point is positioned at the median timing, to detect early reactions whose onset spans the time frame between 30 and 60 min after the transfusion starts, as shown by the graph in Figure 1, and secondly, it presents an optimal time-point that enables a cost-effective deployment of nursing manpower, as compared to the more stringent monitoring at 15 min intervals.

In light of these considerations, 3 time-points from the existing protocol were revised from the existing 10 time-points to 7 time-points. The key differences between the current and proposed protocols are reflected in Table 5. As described earlier, these modifications were made to streamline vital signs monitoring during blood transfusions. However, the measured constituents will remain unchanged—patients will be assessed for their (i) blood pressure (BP); (ii) heart rate (HR); (iii) respiratory rate (RR); (iv) oxygen saturation (SpO_2_); and (v) temperature at each time-point. These will be evaluated against the universally accepted vital signs normal ranges (BP 90/60–120/80 mmHg; HR 60–100 beats per minute; RR 12–20 breaths per minute; SpO_2_ ≥ 95%; temperature < 37.5 degrees Celsius) to ensure that the slightest deviation is captured [33,34].

Our analysis also revealed certain patient characteristics that appeared to predispose them to a higher occurrence of transfusion reactions. These primarily included males aged 50 to 70 receiving packed red cells in oncology wards, who had an elevated body temperature and subnormal blood pressure prior to the blood transfusion. Such data concur with an existing study that reported individuals who had leukopenia (<5 × 10^3^/µL), were febrile or had raised diastolic blood pressure to be at risk of acute transfusion reactions in a retrospective cohort study involving 44,691 events in a Taiwanese hospital [35]. Our findings, revealing the susceptible age and gender subgroups, mirrored an Indonesian study as well [36]. Nevertheless, the identified features in this retrospective review are only exploratory, given the relatively small transfusion reaction numbers (n = 72), which could have skewed and inflated the representation of specific subgroups in the analyses.

On a separate note, the proposed reduction in unnecessary checks by three time-points from the original of ten may bring about several potential benefits to both healthcare professionals and patients. The most direct benefit in an ideal hypothetical scenario is the amount of time saved that can be channeled to other clinical activities without compromising patient safety. This would not only ensure adequate monitoring but also avoid being overly intrusive, which may be more sustainable for nurses in the long run.

### 4.2. Expert Panel Consultations

To ensure that the proposed changes to blood transfusion frequency monitoring were clinically sound and feasible, the team, consisting of a hematologist, registered nurses and a medical laboratory scientist, deliberated extensively. This process was necessary to ensure the patient-first approach was of the utmost priority. The input gathered from these discussions highlighted several core issues. First, care should be appropriately resourced according to patients’ clinical needs and it should take precedence over the convenient reduction in frequency that is purposed to encourage compliance. In other words, additional vital sign checks should be performed if deemed necessary, according to the healthcare professional’s discretion. Second, the team need to empower patients to be able to recognize and report the abnormalities and discomfort experienced in between the monitoring intervals, and this will require proper, rigorous patient education prior to the procedure. Third, nurses need to continue to maintain the rigor and quality of monitoring standards and requirements, as increasing the interval between time-points is not intended to diminish the importance of timely checks of any abnormalities. Beyond human factors, machines for vital signs monitoring must also be readily available to facilitate adherence to the new vital signs monitoring regimen. These insights form a foundation for implementing changes that balance patient safety with operational efficiency. These modifications can then streamline clinical workflows while allowing vigilance for potential adverse reactions, addressing the aim of the paper to determine an appropriate monitoring frequency for blood transfusion monitoring that is able to balance efficiency and safety.

Following the consensus of the proposed changes, senior nursing leaders of the oncology units, where the proposed pilot trial of the reduced frequency of vital signs monitoring would be conducted, were briefed. As the feedback received from them was generally positive, no refinements were made to the modified seven time-point vital signs monitoring frequency.

### 4.3. Potential Challenges and Possible Solutions

While the relaxation of vital signs monitoring frequency during blood transfusion is a calculated and evidence-supported move, one issue that might possibly arise is the delayed detection of deterioration. The proposed frequency sought to strike a balance between timely detection of reactions versus efficient use of nursing manpower. Input from stakeholders was sought: for example, pediatricians. Except for neonatology, which maintained its own protocol frequency, the revised frequency was deemed acceptable by the majority of stakeholders. That said, a multi-pronged safeguard could be incorporated to pair vital signs checking with physical assessments to compensate for the lowered frequency [37]. Future large-scale, in-depth studies determining the variables that are predictive of transfusion reactions and the onset of the respective complications will be necessary to ascertain its effectiveness and refinement.

### 4.4. Significance of Study

The proposed revisions to blood transfusion vital signs monitoring bear significant implications for the transformation of clinical practice among nurses. In many settings and instances where continuous vital signs measurement and documentation is not the default, timely reviews of common procedures that impose a substantial workload on nurses will be beneficial. This paves the way for improvements in other workflows, and at the same time, ensures that current practices keep abreast of scientific advancements.

### 4.5. Study Limitations

The proposed monitoring protocol must be applied with caution, due to several study limitations. Firstly, the review undertaken was a generic and non-systematic one. Secondly, the data analyzed in the retrospective study were from a single institution only. Transfusion reaction numbers were not huge; hence, findings were mostly suggestive and not conclusive. Also, this “relaxed” monitoring frequency should also be assessed for its suitability among those who are younger in age or have cognitive and speech impairments.

## 5. Conclusions

Although most healthcare institutions, locally and elsewhere, adopt a 3 time-point vital signs monitoring frequency throughout blood transfusion, our institutional findings suggest that 7 time-points may be more optimal than 3 or 10 time-points, with special attention given to certain demographic and clinical groups who are at risk of transfusion reactions. This significant modification from the original 10 time-point protocol needs to be evaluated in a pilot. At the same time, it represents an important step in devising the best clinical practices, which can potentially reduce healthcare providers’ workloads while maintaining high standards of patient care.

## Figures and Tables

**Table 1:** Findings on vital signs monitoring frequency, based on online resources (n = 10).

Guideline/ Literature	Country	Year	Level of Evidence ^!^	Monitoring Frequency			
				Pre-BT	During	Post-BT	Total
Southern District Health Board Blood Policy/Health Otago [21]	New Zealand	2014	IV	Yes	15 min after initiation; temperature at 30, 60, 90, 120, 150, 180, 210, 240 min BP at 15, 75, 135, 195 min	Yes *	VS = 3 T° = 10 BP = 6
New Zealand Blood Service [22]	New Zealand	2019	IV	Yes	15 min after initiation; 30 min to 1 h observations thereafter	Yes, within 1 h	3
American Association of Blood Bank (17th Edition) [26]	USA	2011	IV	Yes	5 to 15 min after initiation	Yes *	3
National Institute for Care Excellence (NICE): Blood transfusion NG24 [25]	UK	2015	IV	Yes	Yes, but did not specify interval and frequency	Yes *	Unsure
New York State Council on Human Blood and Transfusion Services/New York State Board for Nursing Guidelines [27]	USA	2012	IV	Yes	15 min after initiation	Yes *	3
Joint UK Blood Transfusion and Tissue Transplantation Services Professional Advisory Committee (JPAC) [23]	UK	2014	IV	Yes	15 min after initiation	Yes, within 1 h	3
HealthDirect Australia/Ausmed Education [29]	Australia	2024	IV	Yes	15 min after initiation, follow organization policy	Yes *	3
London Health Sciences Centre [24]	Canada	2025	IV	Yes	15 min after initiation	Yes *	3
Cortez-Gann et al. Retrospective cohort study [28]	USA	2017	III	Yes	15 min after initiation	Yes, within 1 h	3
Sullivan et al. Literature review [30]	-	2015	V	Yes	15 min after initiation	Yes *	3

VS: vital signs; T°: temperature; and BP: blood pressure. * Article states to monitor vital signs at the end of transfusion but did not specify the frequency. ! Level of evidence is based on the John Hopkins hierarchy of evidence [31].

**Table 2:** Findings on vital signs monitoring frequency during blood transfusion, based on local institution practices (n = 8).

Healthcare Institutions	* Vital Signs Monitoring Time-Point						Monitoring Frequency
	Pre-BT	1st h	2nd h	3rd h	4th h	Post-BT	
Hospital A	Yes	at 15 min; observe for 5 min	Regular check on patient but no vital signs measurement required			Yes	3
Hospital B	Yes	at 15 min	at 75 min	at 135 min	at 195 min	Yes	6
Hospital C	Yes	at 15 min	at 75 min	at 135 min	at 195 min	Yes	6
Hospital D	Yes	at 15, 30, 45, 60 min	at 120 min	at 180 min	at 240 min	Yes	9
Study Hospital	Yes	at 15, 30, 45, 60 min	at 90, 120 min	at 180 min	at 240 min	Yes, within 1 h	10
Hospital E	Yes	at 15, 30, 45, 60 min	at 90, 120 min	at 180 min	at 240 min	Yes, within 1 h	10
Hospital F	Yes	at 15, 30, 45, 60 min	at 90, 120 min	at 180 min	at 240 min	Yes, within 1 h	10
Hospital G	Yes	at 15, 30, 60 min	at 90 min	at 150 min	at 210 min	at 270, 330, 390, 450	11

Pre/Post-BT: pre/post-blood transfusion. * Based on duration of 4 h transfusion.

**Table 3:** Prevalence and characteristics of blood transfusion reactions in 2021–2022.

Outcomes Examined	n (%)
**Total units of blood transfused**	48,362
**Incidence rate of blood transfusion reaction** (overall) Year 2021 Year 2022	72 (0.15) 27 (0.11) 45 (0.18)
**Gender** Female Male	29 (40.3) 43 (59.7)
**Age** Mean; range Below 40 years old 41–50 years old 51–60 years old 61–70 years old 71–80 years old > 80 years old	54.0; 6.0–91.0 19 (26.5) 9 (12.5) 15 (20.8) 13 (18.0) 10 (13.9) 6 (8.3)
**Nature of side-effect** Non-serious ^1^ Serious ^2^	57 (79.2) 15 (20.8)
**Department in which transfusion reaction occurred** Cardiology Emergency Department/Accident and Emergency Intensive Care Unit General Medicine Obstetrics and Gynecology Oncology Orthopedic Operating Theater Pediatric Pediatric Intensive Care Unit General Surgery	5 (6.9) 4 (5.6) 7 (9.6) 12 (16.7) 1 (1.4) 24 (33.3) 9 (12.5) 2 (2.8) 3 (4.2) 2 (2.8) 3 (4.2)
**Blood product(s) with transfusion reaction(s)** Red Blood Cells Platelets Fresh Frozen Plasma Multiple Blood Products	40 (55.6) 23 (31.9) 4 (5.6) 5 (6.9)
**Time to blood transfusion reaction** 0–15 min >15–30 min >30–45 min >45–60 min >60–75 min >75–90 min >90–105 min >105–120 min >120–135 min >135–150 min >150–165 min >165–180 min >180–240 min >240–300 min >300 min	Missing n = 2 (2.8) 1 (1.4) 5 (6.9) 6 (8.3) 9 (12.5) 5 (6.9) 8 (11.2) 4 (5.6) 4 (5.6) 2 (2.8) 2 (2.8) 6 (8.3) 3 (4.2) 7 (9.6) 6 (8.3) 2 (2.8)

^1^ Non-serious: allergic or hypersensitivity reaction and febrile non-hemolytic transfusion reaction. ^2^ Serious: transfusion associated circulatory overload, hemolytic reaction, anaphylactic/severe allergic reaction, acute lung injury (TRALI) and dyspnea. Note: missing data (n = 2; platelet = 1, RBC = 1) were omitted from the analysis.

**Table 4:** Patient characteristics associated with blood transfusion reaction(s).

Reaction Type	Non-Serious (n = 57)	Serious (n = 15)
Gender	Male (n = 34, 59.6%)	Male (n = 9, 60.0%)
Age group	61–70 years old (n = 11, 19.3%)	51–60 years old (n = 5, 33.3%)
Department	Oncology (n = 17, 29.8%)	Oncology (n = 7, 46.7%)
Transfused blood product	RBCs (n = 32, 56.1%)	RBCs (n = 8, 53.3%)
Time to reaction	In the second hour (n = 19, 33.3%)	Within the first hour (n = 4, 26.7%)
Symptoms	Pre-transfusion fever ≥ 37.5 (n = 10, 76.9%)	Pre-transfusion fever ≥ 37.5 (n = 3, 23.1%)
	Pre-transfusion SBP < 90 mmHg (n = 0, 0.0%)	Pre-transfusion SBP < 90 mmHg (n = 1, 100%)

Note: numbers presented here represent the majority proportion only. RBCs: red blood cells. SBP: systolic blood pressure.

**Table 5:** Differences between existing and proposed protocol for frequency of vital signs monitoring.

Time-Points	* Time After Start of Transfusion	
	Before—Existing Protocol	After—Proposed Protocol
T1	Before transfusion (Baseline—pre)	Before transfusion (Baseline—pre)
T2	Time at 15 min	Time at 15 min
T3	Time at 30 min	Time at 45 min
T4	Time at 45 min	Time at 105 min
T5	Time at 60 min	Time at 165 min
T6	Time at 90 min	Time at 225 min
T7	Time at 120 min	Time > 225 min (1 h post-transfusion)
T8	Time at 180 min	**-**
T9	Time at 240 min (End of transfusion)	**-**
T10	Time > 240 min (1 h post-transfusion)	**-**

* Timing is based on 4 h of transfusion.

## Data Availability

The datasets presented in this article are not readily available, due to institutional policy. Requests to access the datasets should be directed to the National University Hospital.

## Public Involvement Statement

No public involvement in any aspect of this research.

## Guidelines and Standards Statement

This study primarily involves a review of guidelines and retrospective data analysis, with the aim of developing an evidence-based protocol for blood transfusion monitoring. Our work represents the foundational research and protocol development stage, which precedes any implementation efforts. While no single reporting guideline perfectly fits our multi-component study design, we have adhered to relevant elements of the STROBE (Strengthening the Reporting of Observational Studies in Epidemiology) guidelines where applicable, particularly for reporting aspects of our retrospective data analysis. Our reporting aims to provide a clear and comprehensive account of the protocol development process, setting the stage for subsequent implementation phases. We have followed the general principles of transparent and comprehensive reporting throughout our manuscript. This includes clear descriptions of our methods for guideline review, data collection and analysis and protocol development. We have strived to provide sufficient detail to ensure reproducibility and to contextualize our findings within the existing body of literature on blood transfusion monitoring. Where relevant to our study design, we have incorporated key reporting elements, such as clearly stating the study design, describing the setting and relevant dates and outlining our analytical approach. We have adapted these principles to fit the unique aspects of our study, including the guideline review and protocol development components that are not typically covered by observational study guidelines. We acknowledge that some elements that are typically included in observational studies, such as detailed participant selection criteria, comprehensive variable definitions and explicit discussion of all potential sources of bias, may not be fully elaborated in our current manuscript, due to its focus on guideline review and protocol development. However, we have aimed to address the potential limitations and sources of bias, particularly in our retrospective data analysis, to the possible extent within our study design.
